# *PAX2* Mutation-Related Renal Hypodysplasia: Review of the Literature and Three Case Reports

**DOI:** 10.3389/fped.2021.765929

**Published:** 2022-01-11

**Authors:** Yu-Ming Chang, Chih-Chia Chen, Ni-Chung Lee, Junne-Ming Sung, Yen-Yin Chou, Yuan-Yow Chiou

**Affiliations:** ^1^Department of Pediatrics, College of Medicine, National Cheng Kung University Hospital, National Cheng Kung University, Tainan City, Taiwan; ^2^College of Medicine, Institutes of Clinical Medicine, National Cheng Kung University, Tainan City, Taiwan; ^3^Department of Medical Genetics, National Taiwan University Hospital, Taipei City, Taiwan; ^4^Department of Pediatrics, National Taiwan University Hospital, Taipei City, Taiwan; ^5^Division of Nephrology, Department of Internal Medicine, College of Medicine, National Cheng Kung University Hospital, National Cheng Kung University, Tainan City, Taiwan

**Keywords:** congenital anomalies of kidney and urinary tract (CAKUT), *PAX2*, renal coloboma syndrome, renal hypodysplasia, chronic kidney disease (CKD)

## Abstract

Paired box 2 (*PAX2*)-related disorder is an autosomal dominant genetic disorder associated with kidney and eye abnormalities and can result in end stage renal disease (ESRD). Despite reported low prevalence of *PAX2* mutations, the prevalence of *PAX2* related disorders may have been underestimated in past studies. With improved genetic sequencing techniques, more genetic abnormalities are being detected than ever before. Here, we report three patients from two families with *PAX2* mutations identified within 1 year. Two patients were adults with chronic kidney disease and were followed for decades without correct diagnoses, including one with ESRD who had even undergone kidney transplant. The third patient was a neonate in whom *PAX2*-related disorder manifested as oligohydramnios, coloboma, and renal failure that progressed to ESRD within 1 year after birth. The phenotypes of *PAX2* gene mutation were shown to be highly variable, even within the same family. Early detection promoted genetic counseling and guided clinical management. The appropriate time point for genetic study is an important issue. Clinicians must be more alert for *PAX2* mutation when facing patients with congenital kidney and urinary tract anomalies, chronic kidney disease of unknown etiology, involvement of multiple systems, and/or a family history of renal disease.

## Introduction

Paired box 2 (*PAX2*)-related disorder, also known as renal coloboma syndrome, was first clearly described in 1988 (1). The clinical presentations were diverse, including renal abnormalities such as renal hypodysplasia or vesicoureteral reflux (VUR); ophthalmological abnormalities such as optic nerve coloboma or dysplasia; and sensorineural hearing loss, central nervous system (CNS) malformation, hyperuricemia, soft skin, and joint laxity (2). Recently, congenital heart disease and skeletal anomalies were also reported (3).

At the time of publication, only 272 affected individuals from 136 different families are documented in the updated database of *PAX2* mutations (https://www.LOVD.nl/PAX2) (4). However, due to the variable clinical phenotypes and sparse experiences reported by clinicians, prevalence of *PAX2*-related disorders may be underestimated (3, 5). *PAX2* mutation may be associated with isolated kidney disease, as shown in one study in which 2 of 20 patients (10%) with urinary tract malformations underwent kidney transplantation but had no ocular abnormalities (5). In a Japanese study, 38 of 457 patients (6.5%) with congenital anomalies of kidney and urinary tract (CAKUT) possessed the *PAX2* mutation (3). Correct diagnoses are crucial for appropriate clinical management, including treatment of VUR (if present) and/or hypertension to prevent complications of chronic kidney disease (CKD), avoiding nephrotoxic medications or food to preserve renal function, and active surveillance of ophthalmologic abnormalities to prevent vision loss. In addition, the correct diagnosis aids genetic counseling, providing comprehensive information for the family to make medical and personal decisions including family planning and prenatal or postnatal molecular genetic testing of other family members (6). Early diagnosis prenatally prompts timely referral of affected individuals to a medical center capable of and experienced in neonatal care of renal failure. Herein, to highlight the importance of this disease, we review the literature and report three patients with *PAX2* mutations, of whom two were diagnosed in adulthood and one as a neonate, all identified within 1 year in a tertiary medical center in southern Taiwan. We emphasized the importance of extending the indications of genetic testing to incorporate more patients at risk for this mutation.

## Case Report

### Case A

A 19-year-old man was born at full-term with a small-for-gestational age birthweight and had been free of any other significant illnesses. His uncle had end stage renal disease (ESRD) with unknown etiology and was undergoing regular hemodialysis.

**Figure 1:**
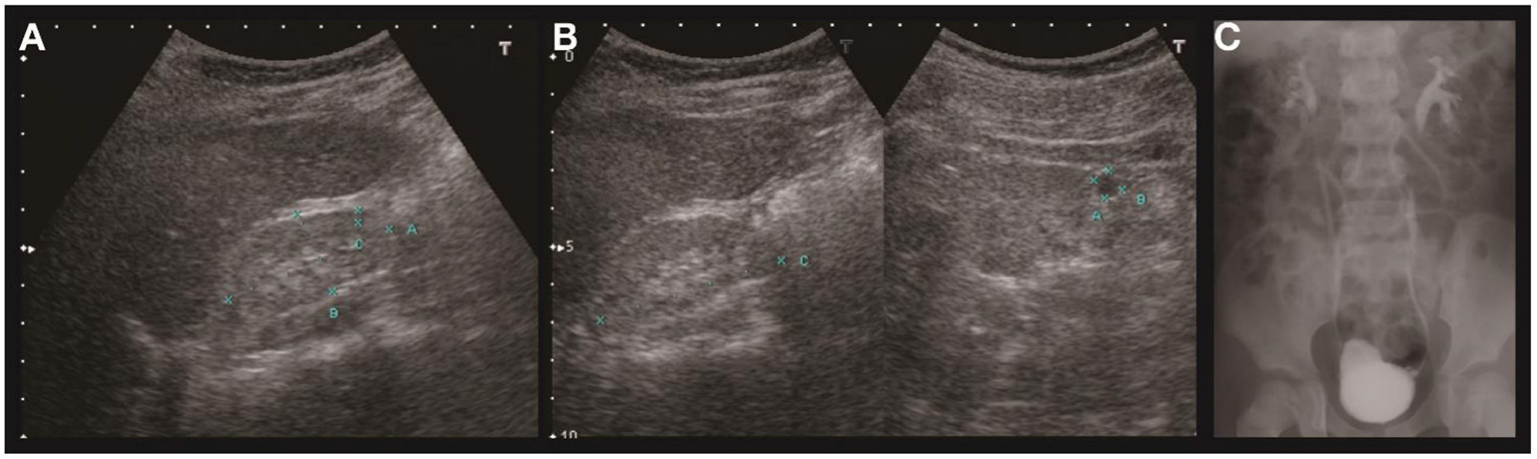
Renal ultrasound and voiding cystoureterography of case A. Sonography of the right **(A)** and left **(B)** kidney revealed hypoplasia and hyperechogenicity of bilateral kidneys. The right kidney measured 4.6 cm in length and left kidney 4.9 cm. A cyst was found in the left kidney. Voiding cystoureterography **(C)** revealed bilateral vesicoureteral reflux (right side grade II, left side grade III).

Proteinuria was found when he was 6 years of age. Lab tests revealed nephrotic range proteinuria (65.4 mg/m^2^/h), elevated blood urea nitrogen (53 mg/dl), and serum creatinine (3.3 mg/dl). Renal sonography and magnetic resonance imaging (MRI) revealed bilateral atrophic kidneys with parenchymal disease and one left renal cyst (Figures 1A,B). Tc-99m-dimercaptosuccinic acid (DMSA) renal cortical scan suggested differential left and right renal function of 45 and 55%, respectively, and cold defects in the upper and lower parts of left kidney. Voiding cystoureterography revealed bilateral VUR, grades II and III at the right and left side, respectively (Figure 1C). He then received bilateral ureteroneocystostomy for VUR at the age of six. Renal biopsy was not performed at that time. The renal function progressed in the following years, and he has received peritoneal dialysis since he was nine and received kidney transplantation at 12 years old.

Not until he was 19 years old were comprehensive examinations carried out to elucidate the nature of his disease, which revealed optic nerve dysplasia, bilateral high-frequency sensorineural hearing loss, short stature, and short digits. Concerning the syndromic presentation, whole exome sequencing (WES) was performed, and results showed a heterozygous *PAX2* mutation c.70dupG (p.L23fs), which was a previously reported pathogenic variant. A heterozygous pathogenic variant of *SLC12A3* (c.539C>A) was also identified, but this mutation was associated with Gitelman syndrome in an autosomal recessive manner, which was not compatible with the presentation of this patient.

### Case B

**Figure 2:**
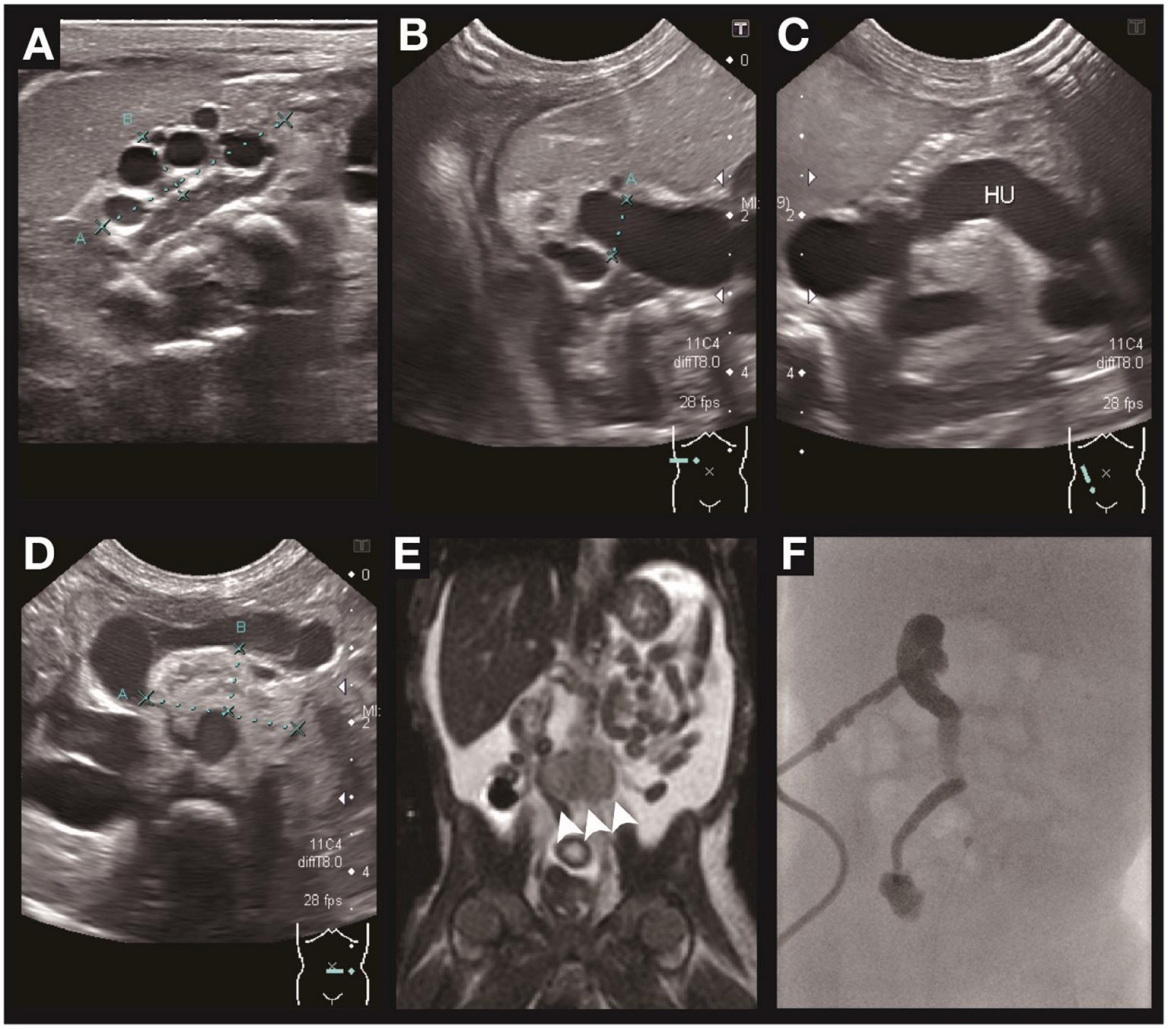
Renal ultrasound, MRI, and antegrade pyelography of case B. Ultrasonography of the urinary systems depicts small size of the right kidney with dysplasia and multiple cysts formation **(A)**, right hydronephrosis **(B)**, right hydroureter **(C)**, and left ectopic kidney with dysplasia and hypoplasia. **(D)** MRI of the abdomen **(E)** confirmed the ectopic left kidney anterior to the aortic bifurcation (arrowhead). Antegrade pyelography **(F)** depicts high grade obstruction at the distal third of the right ureter with contrast medium not passing into the urinary bladder.

A 7-day-old female twin B was born at 33 weeks and 1 day of gestation by Cesarean section because of severe maternal pre-eclampsia. Prenatally, intrauterine growth retardation and oligohydramnios was noted. After birth, she had Potter sequence with flattened nose, micrognathia, and low-set ears. The patient experienced marked body weight gain in the first week of life. The plasma creatinine was 3.51 mg/dl, and the estimated glomerular filtration rate was 3.8 ml/min/1.73m^2^. Renal sonography revealed right dysplastic kidney (Figure 2A) with moderate hydronephrosis (Figure 2B), hydroureter (Figure 2C), and left ectopic hypodysplastic kidney (Figure 2D). Abdominal MRI revealed right-side multicystic dysplastic kidney and left side dysplastic kidney with ectopia anterior to aortic bifurcation (arrowhead in Figure 2E). Antegrade pyelography revealed high grade obstruction at right distal third portion of the ureter (Figure 2F). The ophthalmologic examination showed right optic disc coloboma. WES revealed that she carried a heterozygous *PAX2* mutation c.76dupG (p.V26Gfs^*^28), which was previously reported as a pathogenic variant. A heterozygous mutation of *SALL4* gene (c.2462-2A>G) was also identified on WES, but the patient did not present the related phenotype. Familial genetic testing revealed that the patient had inherited the *PAX2* mutation from her father (reported in case C). Her dizygotic twin sister did not carry the same mutation.

While starting enteral feeding, she had frequent vomiting. Upper gastrointestinal series and abdominal MRI depicted gastric outlet narrowing and obstruction caused by the markedly tortuous and dilated right ureter compressing the antrum of the stomach. Therefore, right ureteral implantation was performed when she was 2 months old. However, vomiting continued postoperatively, and panendoscopy revealed pyloric stricture. She thus received gastroduodenostomy.

The patient's renal function deteriorated progressively and dialysis was started at her 13^th^ day of life. During the hospitalized course, she underwent multiple episodes of infections, including urinary tract infection, peritonitis, and bacteremia. She died in her 9th month from septic shock and multi-organ failure.

### Case C

A 34-year-old man with CKD stage 3, who is the father of case B carrying the same mutation as his daughter, had proteinuria refractory to corticosteroids since he was 5 years old. Renal sonography revealed bilateral renal hypodysplasia and cystic change. Renal biopsy revealed minimal change disease. During routine outpatient surveillance in our hospital, his renal function progressed slowly to CKD stage 3.

The *PAX2* mutation was confirmed by Sanger sequencing after the diagnosis of his daughter (case B). Other findings include bilateral optic nerve dysplasia and hyperuricemia. Despite the identical mutation, he had considerably milder disease than his daughter.

These three cases are summarized in Table 1.

**Table 1:** Summary of the three cases.

**Case**	**A**	**B**	**C**
**Onset age**	**19 years**	**7 days**	**34 years**
**Sex**	**Male**	**Female**	**Male**
**Renal** **presentation**			
Renal morphology	Bilateral hypodysplastic kidneys, left renal cyst	Bilateral hypodysplastic kidneys, left ectopic kidney	Bilateral hypodysplastic kidneys
VUR	Present	Absent	NA
Renal function	ESRD at age 9 years	ESRD at birth	CKD stage 3
**Extra-renal** **presentation**			
Ophthalmology	Optic nerve dysplasia	Right optic disc coloboma	Bilateral optic nerve dysplasia
CNS	NA	Normal	NA
Hearing	Impairment	NA	NA
Other	Short stature, short digits	NA	Hyperuricemia
***PAX2*** **mutation**			
Nucleotide alteration	c.70dupG	c.76dupG	c.76dupG
Zygosity	heterozygous	heterozygous	heterozygous

*CKD, chronic kidney disease; ESRD, end stage renal disease; NA, not attributable; VUR, vesicoureteral reflux*.

## Discussion

*PAX2* gene is one of the most critical genes involved in human urinary system development (7). Although the reported cases are scarce, the prevalence of *PAX2* mutation might be underestimated. One study showed that 10% (2 out of 20) of patients with urinary tract malformation who underwent kidney transplantation possessed the *PAX2* mutation (5). Another study in Japan concluded that 6.5% (38 out of 457) of patients with CAKUT carried the *PAX2* mutation (3). Here, we report three cases of *PAX2*-related disorders identified in our institution to raise alertness of this underestimated mutation. The distinct presentation of two patients (cases B and C) with same genetic mutation loci emphasizes the poor genotype-phenotype correlations (2). Importantly, these case reports demonstrate the difficulties in diagnosis, which led to two of our patients (cases A and C) being identified in the adulthood despite earlier indications of *PAX2*-related abnormalities. In addition to manifestations reported previously, one of our three patients (case B) is complicated by gastric outlet obstruction, which is a feature not reported previously. The present case reports highlight the importance of recognizing the indications for genetic studies when facing patients with CAKUT.

The human *PAX2* gene locates on chromosome 10q24.31 and comprises 12 exons spanning ~84 kb of genomic DNA. It is grouped into four functional domains, including a paired domain, an octapeptide domain, a partial homeodomain, and a transactivation domain (2). *PAX2* is a member of the paired-box transcription factors family that is expressed in the optic vesicles, optic vesicles, mesonephric and metanephric kidneys, spinal cord, midbrain, and hindbrain (2). The *PAX2* gene is especially crucial in the development of the embryonic urinary systems and eyes (8, 9).

*PAX2* plays an essential role in nephrogenesis, especially in the early stage. During the embryonic periods, *PAX2*/*PAX8* activation may be the first signal that guides development of the intermediate mesoderm into the nephric duct (10). In a mouse model, the ureteric bud, which receives inductive signals from metanephric mesenchyme, emerges from the nephric duct at E10.5-E11, and then invades the metanephric mesenchyme (10). Viewed from the molecular aspect, *PAX2* and other transcription factors expressed in the metanephric mesenchyme converge on the ligand glial-cell derived neurotrophic factor (GDNF), which activates RET tyrosine kinase expression in the ureteric bud and induces its outgrowth. Reciprocally, the signals emanating from the ureteric bud initiate the aggregation of the mesenchyme and subsequent conversion to tubular epithelial cells of the nephron (8, 11). During this process, *PAX2* is expressed in molecular structures in the early stage, including the metanephric mesenchyme, ureteric bud, cap mesenchyme, renal vesicles, comma-shaped body, and S-shaped body. Following subsequent development, *PAX2* is downregulated and its expression is rarely detected in the mature collecting duct, and generally absent in glomeruli (2, 12).

*PAX2* gene activity is associated with disease pattern. Under expression of *PAX2* results in hypoplastic kidney and malformed ureter, while overexpression is associated with tumorigenesis (13). To date, around 90 different pathogenic variants of *PAX2* mutation have been found (4). These mutations are mostly located at the paired domain (exons 2–4) and the homeodomain (2). The most common type of pathogenic variant is frameshift mutation, and the most frequently reported mutation is c.76dupG (p.V26Gfs^*^28) mutation (2), which is the mutation noted in the second and the third cases in the present report. Clinically, *PAX2*-related disorder, also known as renal coloboma syndrome, is associated with renal and ophthalmologic manifestations. The disorder is highly penetrant, given that renal disease is identified in 92% of mutated individuals, and eye disease in 77% of individuals (2). The most common renal manifestation is renal hypodysplasia, which is usually bilateral and presented in 65% of involved individuals. Other renal abnormalities include renal insufficiency, cystic kidney disease, vesicoureteral reflux, and other CAKUT. Focal segmental glomerulosclerosis (FSGS) is the most common pathology (3, 6). Eye abnormalities include optic nerve dysplasia and coloboma, which comprise around 72% of patients, and other findings include retinal coloboma, optic nerve cyst, macular abnormalities, or lens abnormalities (2). Reported non-renal and non-ophthalmological manifestations include high-frequency sensorineural hearing loss (7%), short stature, developmental delay, autism, CNS malformations (e.g., Chiari I malformation), hyperuricemia, soft skin, joint laxity, elevated pancreatic amylase, and short digits (2, 6, 14). The case B in our report also presents with gastric outlet obstruction, which we initially attributed to external compression from the tortuous ureter, but pyloric stricture was ultimately diagnosed by panendoscope. However, to our knowledge, there was no previously reported cases of *PAX2* mutation who presented with pyloric stricture. Whether pyloric stricture is a part of the presentation of *PAX2* mutation or it is a coincidence warrants further observation.

We summarize the clinical presentations and mutation types from three recent studies, as listed in Table 2 (3, 14, 15). The median age at initial presentation is 8.2 years, and 41% of cases presented initially between 1 and 10 years of age. Male and female are affected equally. Hypodysplasia is the most common renal abnormality, and the median age of progression to ESRD is 10 years, with a wide range of variation. The most common type of mutation is frameshift mutation, which comprise half of the affected individuals.

**Table 2:** Summary of the clinical presentation and mutation type of previously reported cohort.

	**Yang et al. (15)**	**Rossanti et al. (3)**	**Deng et al. (14)**	**Total**
Case numbers	32	38	10	80
Median age at initial presentation (range)	10 years (prenatal ~ 48 years)	5.5 years (1 month ~ 54 years)	6.4 years (1 day ~ 14.8 years)	8.2 years (prenatal ~ 54 years)
**Age distribution**				
<1 years	7	3	1	11 (15%)
1–9.9 years	14	10	7	31 (41%)
10–19.9 years	8	9	2	19 (12%)
>20 years	7	7	0	14 (19%)
Gender (male/female)	17/15	20/18	4/6	41/39
**Renal presentation**				
Renal hypodysplasia	21	12	10	43 (54%)
Renal insufficiency of unknown cause	9	22	NA	31 (44%)
Cystic kidney disease	4	5	5	14 (18%)
Proteinuria	NA	NA	10	10
Focal segmental glomerulosclerosis	3	3	2	8
Vesicoureteral reflux	4	2	NA	6 (8.6%)
Other	2	2	NA	4
Median age of progression to ESRD (range)	11 years (5–48 years)	7 years (2–11 years)	11.2 years (9.8–16.4 years)	10 years (2–48 years)
**Ophthalmological presentation**				
Optic disc coloboma or dysplasia	6	21	2	29 (36%)
Other	6	4	5	15 (19%)
Normal	8	12	2	22 (28%)
**Non-renal, non-ophthalmological presentation**				
Hearing loss	3	NA	NA	3
Short stature	0	3	1	4 (5%)
**Neurodevelopment**				
Developmental delay	0	2	1	3 (4%)
Autism	0	2	0	2 (3%)
Seizure	2	0	0	2 (3%)
Facial dysmorphism	0	3	0	3 (4%)
Congenital heart disease	0	2	1	3 (4%)
Congenital cystic adenomatoid malformation	0	2	0	2 (3%)
Inguinal hernia	4	0	0	4 (5%)
Other	β-thalassemia, development dysplasia of hip	Retractile testis, polycystic ovarian disease, teratoma, scoliosis	Microcephaly, metatarsal microsomia, teratoma, gout	
**Type of mutation**				
Frameshift	15	21	4	40 (50%)
Non-sense	3	7	1	11 (14%)
Missense	10	7	4	21 (26%)
Splice site	2	2	0	4 (5%)
Insertion	2	0	0	2 (3%)
Deletion	0	1	0	1 (1%)

Among previous studies, no clear genotype/phenotype correlation is observed, and neither the type nor the location of the mutation is predictive of clinical presentation or disease severity (2, 6). However, a recent study has shown a trend that frameshift mutation is more likely to result in typical renal coloboma syndrome, and missense mutation is associated with nephrosis (pathological changes of FSGS without renal morphological abnormalities) (15). Despite this observation, a huge phenotypic heterogeneity is noticed among different patients with the same variants. Even people within the same family harboring the same mutation present with a highly variable disease spectrum, ranging from fatal renal failure at birth to mild renal dysfunction, as shown in the second and third cases in the present report. Briefly, the onset of CKD stage 5 requiring renal replacement therapy ranges from birth to 79 years (2). We list some of the reported family of *PAX2* mutation in Table 3 (2, 16–21), showing an enormous intra-familial heterogeneity on clinical manifestations, including onset age, renal morphology and pathology, age of progression to ESRD, and other extra-renal abnormalities. Further works on genetic investigations are warranted to delineate the relationship between the gene and the clinical presentations.

**Table 3:** Reported familial cases of *PAX2* mutation.

	**Sanyanusin et al. (16)**	**Porteous et al. (17)**	**Ford et al. (18)**	**Fletcher et al. (19)**	**Bower et al. (2)**	**Okumura et al. (20)**	**Iwafuchi et al. (21)**
Case number	4	6	4	3	12	5	3
Onset age	10 weeks ~ childhood	NA	Prenatal ~ 35 years	1.5–21 years	NA	One at 18 years	0–20 years
**Renal presentation** Renal morphology	Three with bilateral renal hypoplasia	Three with bilateral renal hypoplasia, one with cyst, one with nephrolithiasis	Two with bilateral renal hypoplasia	Two with hypoplasia, one normal	Five with bilateral hypoplasia, two with single kidney with hypoplasia	Two with hypoplasia, the other two with malrotation	Two with bilateral renal hypoplasia, one with cysts
Proteinuria	Three with chronic mild proteinuria	NA	No	Two with nephritic range proteinuria, one with mild proteinuria	NA	NA	Proteinuria for all 3 cases
Renal biopsy	NA	NA	NA	NA	NA	One case with glomerulomegaly	One with FSGS
VUR	Three with VUR grade I ~ IV	One with VUR	Two with bilateral VUR grade III	One with bilateral VUR grade III~IV	Three with VUR	NA	NA
Renal function	CKD stage 2~5	Normal ~ CKD stage 5	CKD stage 2~5	CKD stage 4~5	Normal ~ CKD stage 5	CKD stage 2~5	CKD stage 3~5
Age of progression to ESRD	5–15 years	21–35 years	23–60 years	14–24 years	12–79 years	44–61 years	5–7 years
Other			One with nocturnal enuresis				
**Extra-renal presentation** Ophthalmology	All 4 cases with bilateral optic nerve coloboma, one with strabismus and nystagmus	Fiver cases with optic nerve coloboma, the other one with optic nerve pits	Three with bilateral optic disc abnormality, one with absent left macula	All 3 cases with bilateral optic nerve coloboma	Three with small coloboma and small papilla, one normal	All 5 cases with bilateral optic nerve coloboma, one with visual field defect	Bilateral optic nerve atrophy, glaucomatous cupping
Neurodevelopment	One with autism	NA	Normal	NA	NA	NA	Normal
Hearing	NA	NA	NA	One with bilateral sensorineural hearing loss	NA	NA	NA
Other	One with CCAM, two with short stature	NA	One with nystagmus, esotropia	NA	NA	NA	Normal
***PAX2*** **mutation** Nucleotide alteration	c.561del	c.754C>T	c.619insG	c.139-148del	c.223-225dup	c.119_120delGC	c.76dup
Type of mutation	Frameshift	Non-sense	Frameshift	Frameshift	Insertion	Frameshift	Frameshift

*CCAM, congenital cystic adenomatoid malformation; CKD, chronic kidney disease; ESRD, end-stage renal disease; FSGS, focal segmental glomerulosclerosis; NA, not attributable; VUR, vesicoureteral reflux*.

*PAX2* mutation could be associated with isolated renal hypoplasia and asymptomatic or mild disease that may be overlooked by patients and/or clinicians, and thus, the prevalence of *PAX2* mutation may be underestimated (5). The third patient in our present series had mild renal disease and did not receive an exact diagnosis until his daughter was diagnosed. In addition, this mutation may be associated with debilitated and fatal renal disease (2), as shown in case B in this report. Genetic testing for *PAX2* has been suggested in previous reports to be performed among all patients with CAKUT (5). We suggest extending *PAX2* molecular analysis to patients with CKD of unknown etiology, involvement of multiple systems, or a family history of renal disease.

The correct genetic diagnosis is crucial for guiding clinical management and genetic counseling, especially in familial cases. The mainstay of the management is preventing progression and complications of CKD and preserving vision. Strategies include blood pressure control, treatment of VUR, avoidance of nephrotoxic drugs, and/or prevention of retinal detachment (6). Genetic counseling aids the family members on active surveillance of at-risk populations, family planning, and prenatal molecular genetic testing of offspring. As in the familial cases reported here (cases B and C), if the father is diagnosed before the infant is born, the parents might have early genetic counseling and the infant could have been transferred to a medical center earlier for timely care. Early and accurate genetic diagnosis plays a pivotal role in making medical and personal decision for both clinicians and family members.

In conclusion, although previous investigations have shown low prevalence of *PAX2* mutations, we found three cases from two families within the recent 1 year, suggesting that the prevalence of *PAX2* mutation may be underestimated. Due to large variations in the phenotype and severity of disease, genetic testing should be performed in a wide range of clinical circumstances, especially in cases of CAKUT with bilateral renal malformations, syndromic disease with involvement of multiple organ systems, CKD of unknown etiology, or a family history of renal disease.

## Data Availability Statement

The original contributions presented in the study are included in the article/supplementary material, further inquiries can be directed to the corresponding author/s.

## Ethics Statement

The studies involving human participants were reviewed and approved by Institutional Review Board of National Cheng Kung University Hospital. Written informed consent from the participants' legal guardian/next of kin was not required to participate in this study in accordance with the national legislation and the institutional requirements.

## Author Contributions

Y-MC, C-CC, N-CL, J-MS, Y-YCho, and Y-YChi participated substantially in the conception and design of the work, data collection, analysis and interpretation, drafting the article, and critical revisions of the article. All of the authors approved the final version of this paper as submitted and agree to be accountable for all aspects of this work.

## Conflict of Interest

The authors declare that the research was conducted in the absence of any commercial or financial relationships that could be construed as a potential conflict of interest.

## Publisher's Note

All claims expressed in this article are solely those of the authors and do not necessarily represent those of their affiliated organizations, or those of the publisher, the editors and the reviewers. Any product that may be evaluated in this article, or claim that may be made by its manufacturer, is not guaranteed or endorsed by the publisher.
